# Making 'null effects' informative: statistical techniques and inferential frameworks

**Published:** 2018-07-30

**Authors:** Christopher Harms, Daniël Lakens

**Affiliations:** ^1^Department of Psychology, University of Bonn, Germany; ^2^Human Technology Interaction Group, Eindhoven University of Technology, Eindhoven, the Netherlands

**Keywords:** equivalence testing, hypothesis, bayes factors, bayesian estimation

## Abstract

Being able to interpret ‘null effects?is important for cumulative knowledge generation in science. To draw informative conclusions from null-effects, researchers need to move beyond the incorrect interpretation of a non-significant result in a null-hypothesis significance test as evidence of the absence of an effect. We explain how to statistically evaluate null-results using equivalence tests, Bayesian estimation, and Bayes factors. A worked example demonstrates how to apply these statistical tools and interpret the results. Finally, we explain how no statistical approach can actually prove that the null-hypothesis is true, and briefly discuss the philosophical differences between statistical approaches to examine null-effects. The increasing availability of easy-to-use software and online tools to perform equivalence tests, Bayesian estimation, and calculate Bayes factors make it timely and feasible to complement or move beyond traditional null-hypothesis tests, and allow researchers to draw more informative conclusions about null-effects.

**Relevance for patients:**

Conclusions based on clinical trial data often focus on demonstrating differences due to treatments, despite demonstrating the absence of differences is an equally important statistical question. Researchers commonly conclude the absence of an effect based on the incorrect use of traditional methods. By providing an accessible overview of different approaches to exploring null-results, we hope researchers improve their statistical inferences. This should lead to a more accurate interpretation of studies, and facilitate knowledge generation about proposed treatments.

## 1. Introduction

Most scientific research questions are stated in order to demonstrate the prediction that an effect or a difference exists. Does a drug work? Is there a difference between participants treated with antidepressants and patients going to psychotherapy? Common practice is to analyse the resulting studies using null hypothesis significance testing (NHST), for example by performing a *t*-test or a Mann-Whitney-U-test, and to conclude that there is a difference between a control and a treatment group when a difference of zero can be statistically rejected.^1^

There are three scenarios in which the opposite research question, demonstrating the absence of an effect, or the absence of a difference between conditions, might be of interest:

1. Especially in clinical research, it might be important to know if a cheaper or shorter treatment works just as well as a more expensive or longer treatment. Studies designed to answer such questions investigate non-inferiority (e.g., people in one group do not score worse than people in another group) or the statistical equivalence of different treatments (e.g., people in one group score the same as people in another group).

2. We might design a study that has the goal to demonstrate the absence of an effect because we aim to falsify theoretical predictions about the presence of a difference.

3. Even when we do not explicitly aim to test the absence of a theoretically predicted effect, we should be prepared to observe a non-significant finding in any study we perform. Either when examining a novel hypothesis, or when performing a study that was designed to replicate a previous finding, we should be able to statistically evaluate null-results.

In all three cases statistical tools need to be applied that can provide an answer to the question whether we should believe, or act as if, a meaningful effect is absent. As [4] has laid out in his editorial, there is increasing attention to the fact that ‘null results’ need to be published in order to have a coherent scientific body of results. Non-significant results are to be expected, even when examining a true effect, and publication bias (not submitting or publishing non-significant resuls) will inflate effect size estimates in the literature [5,6]. By using statistical approaches that allow researchers to evaluate null-results, researchers will be able to learn more from their data, and publication bias can perhaps be mitigated.

Researchers might want to know if a null-hypothesis is true, and therefore be interested in ‘proving the null’. However, there are no statistical techniques that can unconditionally answer the question whether or not the null-hypothesis is true. As we will see below, statistical techniques that allow researchers to evaluate null results only allow conclusions about the null-hypothesis in relation to some specified alternative hypothesis. The null-hypothesis can not be statistically evaluated in complete isolation. Furthermore, it is impossible in empirical research to ‘prove’ a prediction, since theories and predictions are inherently probabilistic in an inductive empirical science. Rare events will happen, and thus the absence of an effect is always concluded based on a defined probability of making an error, or given a particular level of certainty. The aim of the present article is to give an overview of statistical methods suited to investigate ‘null effects’, and explain how to translate the statistical results from these methods into valid conclusions about the prediction that is tested. We provide a hypothetical example that is analyzed using four different methods, discuss how to interpret the results (as well as possible misinterpretations), and briefly explain which inferential frameworks these different methods are based on.

## 2 Investigating 'null effects'

It is common practice in empirical research to rely almost exclusively on null-hypothesis significance testing to investigate the presence of an effect. Because a null-hypothesis test can only reject the null (i.e. commonly the hypothesis of ‘no effect’), it cannot be used to inform us about the absence of an effect in the population. When we observe a non-significant effect (e.g., *p* *>* ** , where ** is the level of significance chosen ahead of data-collection), all we can conclude is that, assuming the true effect size in the population is zero, the observed effect size was not sufficiently different from zero to reject the null hy-pothesis without, in the long run, being wrong more often than a desired error rate. This does not rule out the possibility that the true population effect size differs from zero. It is also possible that the experiment might have had relatively low power to de-tect the true effect size, or – equivalently – a high probability of making a Type 2 error (not rejecting the null-hypothesis when a true effect is present in the population).

Null-hypothesis significance testing answers a specific ques-tion (i.e., can we reject the null-hypothesis?). One can argue that in most studies without random assignment to conditions, and perhaps even in some studies with random assignment, it can be expected that the true (population) effect size is always unequal to zero. Often an effect size of exactly zero (as assumed in the null hy-pothesis) is implausible [7]. For hypothesis testing, however, it is a useful model for comparison. When another question is of interest (i.e., can we conclude a meaningful effect is absent?), other statistical techniques should be used. Several statistical techniques have been developed to allow researchers to draw meaningful inferences about null-effects. Here, we will discuss equivalence testing, Bayesian estimation (i.e., the ROPE proce-dure) and Bayesian hypothesis testing (i.e., the use of Bayes fac-tors). We will demonstrate these different approaches using a fictional dataset from an imaginary study. Imagine, you want to investigate whether mindfulness meditation has an effect on lower back pain (LBP), which is an increasingly common problem among desk-working adults. In a fictional study patients with lower back pain are recruited and randomly assigned to either an eight week mindfulness meditation class (the treatment group) or an eight week waiting list condition (a passive control group). At the time of inclusion in the study and after the eight week study period self-reported lower back pain intensity is measured on a 100mm Visual Analogue Scale (VAS) [8,9]. The dependent variable to be analyzed is the difference between the VAS scores at the end and start of the study. The mean change over the eight week period between the treatment group and the control group is examined using a two-sample *t*-test.^2^

The sample size of the study needs to be determined based on an *a priori* power analysis. Based on a discussion with experts in the field, the smallest effect size of the treatment that is still deemed worthwhile is Cohen’s *d* = 0.30, and the study is designed to have a high probability of observing a statistically significant effect, if there is a true effect at least as large as this smallest effect size of interest. Assuming it is relatively easy to get people to enroll in the study, and further assuming the researchers want to prevent incorrectly concluding the two treatments differ, the alpha level is set to 0.01 and the desired power for the smallest effect size of interest is set at 90%.^3^ This means that if there is a true effect of *d* = 0.30 or larger, we have at least 90% chance of observing a significant effect (in the long run). Based on the desired error rates, the power analysis indicates 332 patients per group should be enrolled in the study.

**Figure 1.**
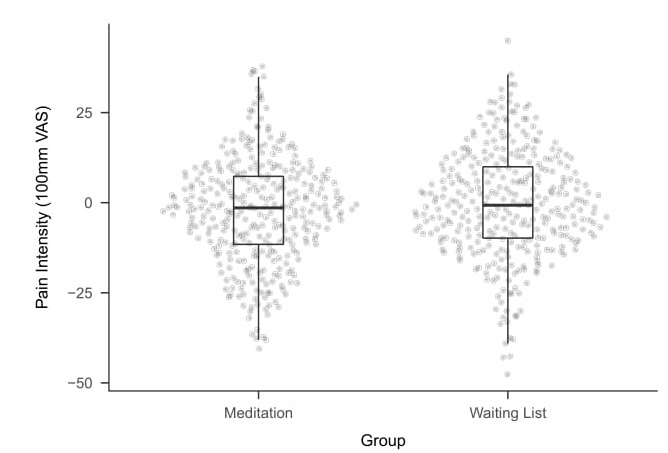
Plot for the data of the imaginary study. Each dot represents a single case. Box plot shows median and 25% and 75% quartiles. Y-axis is dependent variable, i.e. Change in pain intensity after either 8 weeks of meditation class or after 8 weeks of being on the waiting list.

For the imaginary study we simulated random samples using R from two independent normal distributions.^4^ The fictional measurements collected from 664 participants are visualised in Figure 1. The mean change in self-reported lower back pain intensity on the 100mm VAS over the eight week period (and stan-dard deviations) are *−*2.30 (14.77) in the Meditation group and*−*0.39 (15.13) in the control group.

### 2.1 Null-hypothesis significance test

A common first question in experiments where participants are randomly assigned to two conditions is to examine whether we can statistically reject a difference between the groups that is exactly zero. This null hypothesis can be examined by performing a *t*-test with the chosen significance level of ** = 0.01. The two-sample Welch’s *t*-test (which does not assume equal variances) yields *t*(661.63) = *−*1.64, *p* = .101. The *p*-value is not statistically significant, which means the estimated population difference in the data is not extreme enough to reject the hypoth-esis that the true changes in pain scores in both groups are the same. A non-significant test result does not mean that the null hypothesis is true. Non-significant results simply indicate that the data are not surprising if we assume there were no true dif-ferences between the conditions. This might be because there is no difference between the two populations from which the two groups are sampled, in which case a non-significant effect is ex-pected with a frequency of 1 *−* ** = 0.99. But it is also possible that there is a difference, but due to sampling error, it was not observed, which should happen 10% of the time if the true ef-fect size for which we have 90% power is *d* = 0.30 (and more often if the difference between groups in the population is smaller than *d* = 0.30).

It should be noted that there are different frameworks for performing significance tests in frequentist statistics. Statistician Sir Ronald Fisher introduced the concept of significance tests. In the Fisherian test, a *p*-value is computed under a null-hypothesis. Importantly, in the Fisherian significance test no alternative hy-pothesis is specified. Jerzy Neyman and Karl Pearson extended on Fisher’s significance tests (much to Fisher’s dismay) by in-troducing the concepts of power and alternative hypotheses [12]. The goal of Neyman-Pearson significance testing is to warrant long-run error rates. This requires an *a priori* power analysis (as was done above) where an alternative hypothesis is specified and the long-run Type 2 error rate is chosen. In applied practice, a hybrid has evolved that combined aspects of the two paradigms of statistical testing [13]. For proper statistical inferences it is important to use the statistical methods in the formally correct man-ner, in line with the theoretical basis upon which they were de-veloped. In this section and the section on equivalence testing, we focus on the Neyman-Pearson approach of hypothesis test-ing and interpret the results of a statistical test as a dichotomous decision how to act for which we have decided on long-run error rates.

A null hypothesis significance test cannot distinguish between the conclusion that an estimated population difference is too small to be considered meaningful, or an inconclusive result (i.e., the effect is not statistically different from zero, but also not statistically smaller than any effect you care about). This often leads researchers to believe non-significant results are not informative. While a non-significant result in a null-hypothesis significance test *per se* does not allow us to decide between the absence of a meaningful effect, or an inconclusive result due to low power, the data might be informative when analyzed with statistical tests that do allow researchers to draw more useful conclusions about null-effects.

In the past researchers were advised to interpret non-significant results by performing a sensitivity analysis, and report an effect size the study had high power to detect. For example, if a study had 90% power to detect an effect of d = 0.30, researchers might conclude that if there is an effect, it would most likely be smaller than d = 0.30. This is referred to as the ‘power approach’ [14,15]. Based on the absence of a significant effect, researchers would conclude that it is unlikely that a true effect as large or larger than a specific size is present. However, the ‘power approach’ is superseded by the development of equivalence tests [14], and is no longer recommended.

### 2.2 Equivalence tests

There is no statistical procedure that can confirm that the difference between two groups is exactly zero (beyond sampling the entire population, and finding that the observed difference or effect is exactly 0). However, it is possible to test whether an effect is close enough to zero to reject the presence of a meaningful difference. In this approach, researchers need to specify the difference that is considered too small to be meaningful, the smallest effect size of interest (SESOI). The SESOI is in clinical domains also referred to as the ‘minimal clinically important difference’ (MCID). A statistical test (very similar to the traditional t-test) is performed that examines whether we can statistically reject the presence of a difference as extreme, or more extreme, as the smallest difference we care about. If we can reject the presence of a difference (with a desired alpha level) we can act as if the difference is *practically equivalent* to zero. This procedure is known as *equivalence testing* [16].

For clinical scenarios in which pain intensity is measured using a 100 mm VAS in patients with lower back pain, a difference of 9 mm is considered to be a minimal clinically important dif-ference. This is based on the finding that a difference of 9 mm is the point where patients indicate that they subjectively feel ‘slightly better’ instead of ‘equal’ [17]. Note that this is only one approach to determine a smallest effect size of interest, and other justifications for a smallest effect size of interest are possible [18]. Ideally, the SESOI should be informed by theory and previous research (such as meta-analyses or systematic reviews). The SESOI needs to be determined before collecting the data (similar to decisions about the sample size, the alpha level, and the desired statistical power). An informative study should be designed to have sufficient power both (i) to detect an effect that exceeds the SESOI and (ii) to demonstrate equivalence to zero or another specific value (thus rejecting the smallest effect size of interest).

One way to test for equivalence is to perform the Two One-Sided Tests (TOST) procedure. A lower (˜ L ) and upper (˜ U ) equivalence bound is specified (e.g., a difference of − 9 mm or 9 mm on a 100 mm VAS).A first one-sided test is performed to examine whether we can reject effects smaller than ˜ L = − 9 mm, and a second one-sided test is performed to test whether we can reject effect larger than ˜ U = +9 mm. If both one-sided tests are significant, we reject the presence of a difference more extreme than ±9 mm, and conclude that the group difference is statistically equivalent to zero, given the equivalence bounds that were chosen.

**Figure 2.**
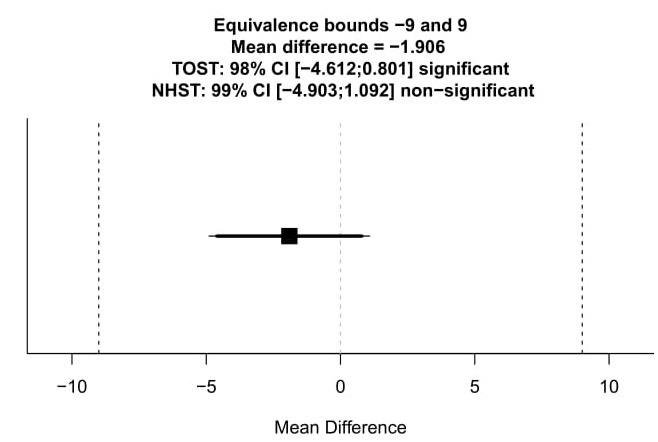
Visual representation of the equivalence test. Plotted is the confidence interval for the mean difference between the two groups. Based on our choice for an ˜ of 0.01 the bold line visualizes the 98% confidence interval used for the TOST approach, while the thin 99% confidence interval is used for the traditinal significance test against the null hypothesis of zero difference. The equivalence test is significant, which can be inferred from the fact that the 98% confidence interval does not overlap with the equivalence bounds of − 9 mm and +9 mm and we can reject the presence of a clinically meaningful effect.

[19] created an R-package (TOSTER) and a spreadsheet to perform equivalence tests for t-tests, correlations, proportions, and meta-analyses. Performing an equivalence test (again using Welch’s t-test) on our fictional data, with an ˜-level of 0.01, yields a significant result (t_1_(661.63)= 6 .11, p< . 001; t_2_(661.63) = − 9.40, p < .001). The result is vizualized in Figure 2, where the 98% confidence interval is plotted and compared to the equivalence bounds of − 9 mm and +9 mm. The width of the confidence interval is 1 − 2˜ since two one-sided tests are performed, both of which need to be significant to conclude equivalence [16]. Using a Neyman-Pearson approach to statistical inference, in which the goal is to make dichotomous decisions while controlling error rates ata desired level, we can *act* as if the difference between the two groups is smaller than the minimal clinically important difference of ±9 mm, without being wrong too often in the long run. It is important to note, that the confidence intervals here are only used to check whether the 98% confidence interval falls within the equivalence bounds.

This is equivalent to performing the two one-sided tests (TOST) explained above. The interpretation of confidence intervals in a frequentist estimation framework has been critically discussed, e.g. by [20].

The present example represents the case of a non-significant result that is equivalent to zero. It should be noted, that the equivalence testing approach also allows for significant and equivalent outcomes: If a much larger sample size had been collected and the same mean difference was observed, the 99% confidence would no longer overlap with zero, which would allow us to reject the null-hypothesis. With both the traditional significance test as well as the equivalence test being significant, we can conclude a mean difference that is statistically different from zero, while at the same time being *practically insignificant*.

Accessible introductions to equivalence testing are available [14,18,19], and equivalence tests can be performed in R, using a spreadsheet [19], or using the free software jamovi. We provide scripts for R [1] and jamovi [3] to reproduce the analyses and results in this paper as supplemental material.

### 2.3 Bayesian estimation

Frequentist statistics, which underly null-hypothesis significance tests and equivalence tests, have the goal to control error rates in the long run. Researchers can’t know whether the conclusion made for any single study is one of these errors. Bayesian statistics allows researchers to make statements about the probability of single events and specific hypotheses, given the observed data because it uses a different understandings of ‘probability’. The debate about which definition of probability is ‘correct’ or more adequate has led to a debate among statisticians and philosophers of science that has been going on for many decades. Luckily, researchers don’t need to choose a side (unless they want to), because both approaches can be used side-by-side when analysing data. Excellent introductions to Bayesian statistics from an applied perspective on statistics can be found in [21] or [22].

Bayesian statistics is best understood in the context of statistical modelling. A statistical model is a mathematical description of the probability of data. In Bayesian statistics a model consists of three different parts. The first part is called a *prior distribution*: For each parameter we choose a probability distribution that describes expectations about possible parameter values. This prior can be understood as our ‘belief’ before seeing the data (hence the *prior*). This terminology already highlights the distinction between the frequentist and the Bayesian understanding of probability: While frequentists consider ‘probability’ as a statement about long-term frequencies of events, Bayesians think of ‘probability’ as a ‘degree of belief’. This subjective interpretation is easily explained – and very intuitive to some – but not without criticism. Even among Bayesians there is disagreement about the subjective nature of the prior. Gelman et al. [23] provides one accessible commentary on this debate.

As the second part of a Bayesian model, we take the observed data into account through a *likelihood function*, and calculate a posterior distribution through the use of Bayes’ theorem. In mathematical notation this is

$$P(\theta |Data)\;=\;\frac{P(Data|\theta )\;\cdot \Pi (\theta )}{P(Data)}$$

where *π*( ) is the prior distribution for our parameter , and *P* (*Data|* ) is the likelihood function of the model. *P* ( *|Data*) is the posterior distribution of the parameter after seeing the data (i.e., the conditional probability of the parameter values given the observed data). The posterior distribution is thus – analogous to the prior distribution – our belief about different parameter values for after having seen the data. When moving from a prior to a posterior distribution credibility is reallocated from the prior distribution to a posterior distribution that represents credibility informed by both the prior information and the data. If the prior distribution is accepted to represent a valid allocation of belief, the posterior distribution represents rationally updated belief through the observed data. The term *P* (*Data*) in the de-nominator is a normalizing constant in order for the posterior *P* ( *|Data*) to be a proper probability distribution. We will later refer to it in the section about Bayes factors as the *marginal like-lihood* of the model (since it is the likelihood marginalized over all parameter values), also called *model evidence*.

Kruschke et al. [24] introduced a pre-defined Bayesian model that can be used to draw inferences about the estimated differences between two independent groups. This procedure provides researchers with a simple and easy-to-use test to evaluate the data in a Bayesian estimation framework. When using a Bayesian sta-tistical model, samples from the posterior distribution are gener-ated which can be used to make inferences about the data. One way to summarise the posterior distribution is to provide inter-vals of parameter values that are considered to be most credi-ble. In Bayesian statistics Highest Density Intervals (HDI) are commonly used. For example, a 89% Highest Density Inter-val contains the values which, based on the statistical model used (including the prior distribution), are considered the 89%most credible. For the pre-defined model by [24] the posterior samples can be generated and summarised using the ‘BEST’ R-package [25] or a web-app [26]. Importantly, even if only sum-maries are presented such as means, standard deviations, or cred-ibility intervals, the whole posterior distribution is available to provide the statistical inference [27].

**Figure 3.**
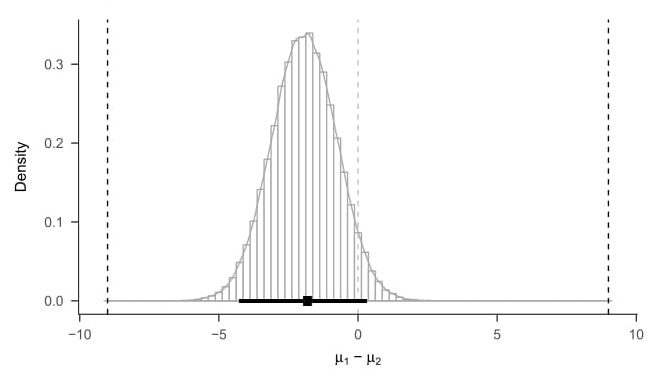
Histogram with superimposed density estimate of samples from posterior distribution for the Bayesian *t*-test model [24]. Thick bar is the 95% Highest Density Interval, indicating the 95% most credible values for the mean difference between the two groups. The square in the interval is the Maximum A Posteriori estimate, i.e. the most credible value from the posterior distribution.

In our imaginary study where we compare an 8-week meditation class to patients on a waiting list we find a 95% posterior Highest Density Interval (HDI) of [*−*4.24; 0.32] for the difference in pain intensity between the two conditions. This means that the 95% most credible values for the difference in means, given our model, which incorporates both the prior information and the observed data, lie between *−*4.24 mm and 0.32 mm. Figure 3 visualizes this result.

Some differences between the confidence interval reported above and the Bayesian HDI are to be expected. The prior affects the width and location of the HDI in Bayesian estimation, and whenever the priors that are used for the model are not uniform, an HDI and a confidence interval will differ to a certain extent. With sufficient information from the observed data, the collected data will outweigh the prior, but with smaller amounts of data, it can be advisable to explore the impact of different priors on the inference. In the BEST model, the priors are not uniform but chosen to have minimal impact on the inferences, so even if the number of observations is relatively small, the prior should not have too much influence on the results [24].

The posterior distribution can be used to answer several other questions as well. Besides the HDI, we can find the most credible value for the difference between the two groups, which would be the posterior mode, or *Maximum A Posteriori (MAP) estimate*, which is *−*1.81 (and differs slighty from the frequentist estimate of the difference due to the prior). When one aims to make a dichotomous decision about parameter values based on the posterior distribution, Kruschke et al. [27] propose to define a *region of practical equivalence* (ROPE) which is identical to setting equiva-lence bounds based on a smallest effect size of interest as laid out above. The ROPE procedure uses the following decision rule [28]:

If the 95% HDI of the [parameter’s posterior distribution] falls completely outside the ROPE than reject the null value, because the 95% most credible values of the parameter are all not practically equivalent to the null value. If the 95% HDI of the [parameter’s posterior distribution] falls completely inside the ROPE then “accept” the null value for practical purposes, because the 95% most credible values of the parameter are practically equivalent to the null value. Otherwise remain undecided.

By comparing the 95% HDI with the region of practical equivalence from ∆_*L*_ = *−*9 mm to ∆_*U*_ = +9 mm, based on the same equivalence bounds as before, researchers can conclude equivalence when the HDI lies within the region of practical equivalence (or between the equivalence bounds). Because the 95% HDI ([*−*4.24; 0.32]) lies well within those bounds (as can be seen in Figure 3), we declare a difference of exactly zero to be accepted for practical purposes based on the decision rule above. We do not, however, accept or reject any other specific value within the ROPE. In the vocabulary of Bayesian statistics, using a decision rule on a posterior distribution of a single model does not constitute 'hypothesis testing'. The term 'Bayesian hypoth-esis testing' refers strictly to the use of Bayes factors for model selection, which we will discuss in the next section. An alter-native way to investigate practical equivalence using a Bayesian posterior distribution would be to examine the probability mass contained in the ROPE [29]. It is important to highlight that the basis for inference is the full posterior distribution. Thus, it is up to the researcher to decide whether they want to make a dichotomous decision about a single parameter value or rather make a probability statement (see Discussion).

The Bayesian ROPE procedure is quite similar to equiva-lence tests, but there are several important dinstinctions. In the Bayesian approach we can make statements about which values we believe are most credible, based on the data and the model, while in frequentist statistics we make dichotomous decisions based on long-run error rates. Frequentist statistics is concerned with frequencies of events in the long run. Null-hypothesis significance tests and equivalence tests as discussed previously aim to control the rate at which incorrect conclusions are drawn about the presence or absence of effects at pre-specified levels. As a consequence, the width of a confidence interval is directly re-lated to the chosen level. In the Bayesian approach, on the other hand, no statements about rates of decision errors can be made without additional assumptions and analyses. Kruschke et al. [27] use a 95% interval because of the convention to set the significance level at 5%, but the width of the HDI should only be seen as a useful summary of the complete posterior distribution, and is not related to the 5% Type 1 error rate of the confidence interval.^5^

### 2.4 Bayesian hypothesis testing with Bayes factors

The ROPE procedure uses Bayesian statistics to estimate the parameter values that are most credible and then uses a decision rule to accept or reject specific values. Bayesian statistics can also be used to directly test two competing models. Hypothesis testing can be considered as a special case of model selection, where two specific hypotheses are expressed in terms of competing models. One way to perform this type of model selection in Bayesian statistics (or Bayesian hypothesis testing) is to compare the marginal likelihoods of two models *M*_0_, the null model, and *M*_1_, the alternative model, and quantify the relative model evidence in terms of a ratio: 

$${\text{BF}}_{01}=\frac{P(Data|M_{0})}{P(Data|M_{1})}$$

This ratio is called a Bayes factor and allows statements about relative model evidence. A Bayes factor of BF_01_ = 4.2 can be interpreted as ‘the data provide 4.2 times more evidence for *M*_0_ than for *M*_1_’.^6^ Bayes factors indicate by what amount the relative belief in the models should shift according to rational Bayesian belief updating:

$$\underset{\text{Posterior Odds}}{\underset{︸}{\frac{P(M_{0}|Data)}{P(M_{1}|Data)}}}\;=\;\underset{\text{Prior Odds}}{\underset{︸}{\frac{\text{Π(}M_{0}\text{)}}{\text{Π(}M_{1}\text{)}}}}\times \underset{\text{Bayes factor}}{\underset{︸}{\frac{P(Data|M_{0})}{P(Data|M_{1})}}}$$

The most common approaches to calculating Bayes factors model the null-hypothesis as a point, with an alternative model that distributes the probability of the true value across a range of possible values. This choice for a null-model is generally similar to frequentist hypothesis testing, where the null hypothesis is commonly also a point hypothesis of exactly zero. For Bayes factors that closely resemble traditional statistical tests, the two competing models are distinguished by different prior distributions for a parameter (usually a test statistic). Defining a reasonable alternative model is an important part of calculating a Bayes factor. There are different ways in which the alternative model can be specified. One way is to use researchers’ beliefs or expectations of theoretical predictions. Another way would be to use data observed in previous studies to inform the alternative model [31, 32].

**Figure 4.**
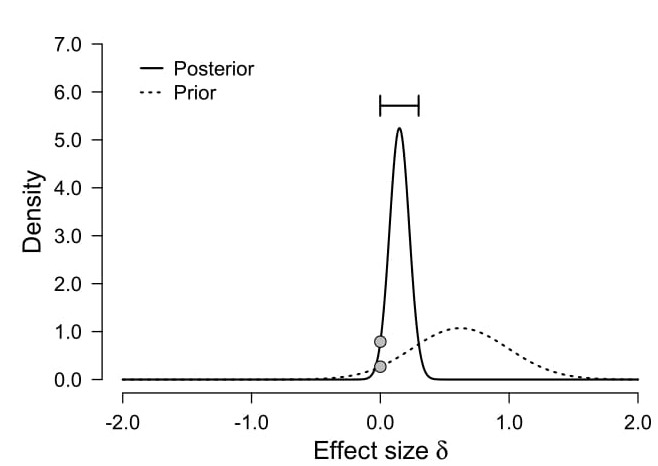
illustrates the two models compared when calculating a Bayes factor. In the figure *M*_0_ is represented by a point-null hypothesis and *M*_1_ is represented by a distribution that assumes small effect sizes are more likely than large effect sizes, but which is not very restrictive and assigns probabilities to a wide range of possible values.

**Figure 4.** Visual representation of the Bayes factor as Savage-Dickey ratio [37]: The Bayes factor can be understood as the ratio between the posterior and the prior at = 0 (indicated by the two grey dots).

A common criticism on Bayes factors is that they are much more sensitive to the specification of the prior than Bayesian model estimation. While the data quickly overwhelms the prior in a Bayesian estimation framework (such as the ROPE procedure), the priors in a Bayes factor have much more weight. It is important to note, however, that priors have different purposes in the two approaches: In Bayesian models for estimation, the priors are used as a device for regularization and shrinkage of parameter estimates. This can be driven by subjective beliefs or statistical considerations (see discussion on subjective and objective use of priors above). For Bayes factors, on the other hand, priors should represent the predictions of a theory. Therefore, researchers have cautioned against the use of ‘default’ priors when calculating Bayes factors [33], which are a compromise between general expectations about effect sizes and useful mathematical properties [34], but these default model specifications should only be chosen if they actually reflect a useful alternative model given the research question. Moreover, Bayes factors – very much like *p*-values – do not convey information about the magnitude of an effect or the uncertainty in its estimation. See [35] for additional criticisms on Bayes factors.

Bayes factors can be used to examine null effects by quantifying the relative evidence in the data for a null-model compared to an alternative model. In the Bayes factor calculation for our hypothetical data we wanted the prior for the alternative model to represent our expectation about the presence of a true effect. If our 8-week meditation class reduces pain intensity on a 100mm VAS scale compared to the active control condition, we expect it to be similar in size to other non-pharmaceutical interventions. Hoffman et al. [36] performed a meta-analysis of different psychological inter-ventions on pain intensity in patients with chronic lower back pain, and provided an estimated meta-analytical effect size of *d* = 0.62 (95% CI: [0.25; 0.98]) when comparing the effect of cognitive-behavioral therapy (CBT) against a waiting list con-dition. Therefore, we calculate a Bayes factor based on the expectation that a mindfulness meditation intervention might have a similar effect size.

We specify an alternative model with a normal prior distribution centered on 0.62 with a standard deviation of 0.37 (calculated from the confidence interval): *M*_1_: *ẟ*N(0.62,0.37). The M_1_ model is compared against the null model *M*_0_ with a prior that has its point mass at 0 (i.e. a point null hypothesis).

A Bayes factor for the *t*-test from our example study yields *BF*_01_ = 2.95 [38]. We can thus conclude that the data is 2.95 times more in favour of the null model compared to the informed alternative model that we specified. The Bayes factor can be represented visually as in Figure 4: It shows the ratio between the height of the prior and the height of the posterior distribution at ẟ=0, the point of interest for the null hypothesis. This ratio is called the Savage-Dickey ratio [37]. Although Bayes Factors can be interpreted as a continuous measure of model evidence, thresholds for inter-preting Bayes factors have been proposed [39], which might be useful for researchers who begin to report and interpret Bayes factors. A Bayes factor of 1 indicates the data are equally likely under both models. Bayes factors between 1 and 3 constitute mere ‘anecdotal’ evidence, which is considered ‘worth not more than a bare mentioning’ [39]. Thus, although the data support the null model over the alternative model specified by the prior, there is no good reason to conclude in favor of either model – at least if not either model is much more reasonable than the other *a priori* without respect to the data (we extend the discussion on prior belief in each model below). Stronger model evidence would be desirable, which means more data need to be collected [40].

**Figure 5.**
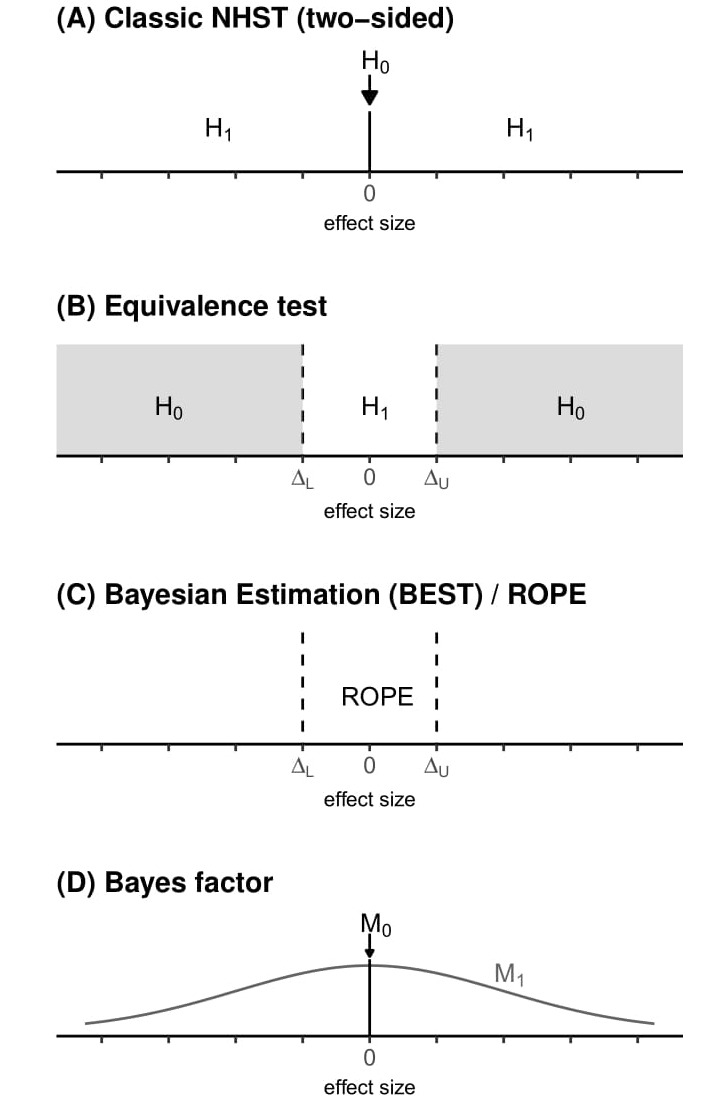
Illustration of the different hypotheses under investigation [18]. **(A)** The classic two-sided significance testing aims to reject a point null hy-pothesis (here an effect size of exactly zero). **(B)** In equivalence test, the *H*_0_ of no equivalence is tested (grey region), so the white area is the rejection region. **(C)** For the Bayesian estimation approach, the 95% highest density interval of the posterior is compared against the Region of Practical Equiv-alence (ROPE) between ∆_*L*_ and ∆_*U*_ . **(D)** For the Bayes factor, two models are compared that differ in their prior distributions: The*M*_0_ prior is a point mass of 1 at an effect size of 0, the alternative model *M*_1_ is here plotted as a Normal distribution as an example. Note, that other alternative models can be used, e.g. centered on a value derived from theory or previous studies (see Figure 4).

The difference between the result of the Bayes factor analysis, the equivalence test, and the ROPE procedure reported earlier has several reasons. Most importantly, the questions that were asked differed across the tests. The equivalence test sought to reject an effect specified by and upper and lower equivalence bounds of ±9 mm (see Figure 2), and the ROPE procedure examined wether the 95% HDI fell within the region of practical equivalence (Figure 3). The Bayes factor investigated the data was more in line with a null model or an alternative model specified based on expectations derived from previous studies. Researchers need to be aware of the precise question they want to ask from the data and the method they use to do answer their question. In order to draw informative inferences from the data, it is crucial that a statistical test is selected in which alternative hypotheses are defined that answer a question of interest.

The Bayes factor tells us how much our belief in the null model versus the alternative model should change. It does not, however, directly tell us how likely the null hypothesis is, be-cause it is a relative measure. As can be seen in the equation above, to calculate the posterior odds of the two competing hypotheses, a researcher needs to combine the Bayes factor with prior probabilities for the two hypotheses. There is rarely an ob-jective answer to the question of prior odds, and researchers are free to hold different beliefs. If we feel that the two models are equally likely *a priori*, i.e. the prior odds are 1:1, the Bayes fac-tor would be equal to the posterior odds. If, on the other hand, we feel that the null hypothesis is four times more likely than the al-ternative hypothesis (before seeing any data from the study) and the Bayes factor is *BF*_01_ = 2.95, we should believe that the null model is about 11.78 (4 times 2.95, with a small difference due to rounding) more likely than the alternative after seeing the data. Since different researchers can have different beliefs about the prior odds of two hypotheses, Bayes factors are commonly reported without a reference to prior or posterior odds and the reader is assumed to update their own priors. If a researcher accepts the prior distributions for the parameters in the models compared in the Bayes factor, the Bayes factor contains the necessary information to update their own prior odds and make an inference – but the Bayes factor is by itself not sufficient to reach a conclusion. Prior odds are a necessary part of the inferential method when using Bayes factors.

## 3 Discussion

There are good reasons wanting to test whether meaningful effect sizes or theoretically predicted differences are absent in data that have been collected to examine a hypothesis. In recent years, statistical techniques such as equivalence testing, Bayesian estimation, and Bayesian hypothesis tests have become more widely available through open source software tools such as R [1], jamovi [3], and JASP [2], and accessible introductions with detailed examples [18,21,22]. These statistical tools allow researchers to move beyond merely testing whether the null hypothesis can be rejected in a null-hypothesis significance test. These complementary statistical approaches invite researchers to more carefully consider and specify which effect sizes they predict when there is a true effect. A statistical evaluation of the observed data should allow for informative conclusions about null effects, and when planning a study and performing statistical inferences researchers should more explicitly consider the possibility that the null hypothesis could be true. This implies that an informative study should be designed that allows one to draw conclusions about both the presence and the absence of a meaningful effect. We hope that the use of correct statistical approaches to evaluate null-results will prevent the common mistake to interpret a *p*-value larger than the alpha level (e.g., *p > .*05) as the absence of an effect.

In the context of clinical trials, the repeated use of equivalence and non-inferiority tests can have negative effects on the conclusions derived from such research. That is, if sampling and measurement error are large and the equivalence region is rather wide, repeated studies comparing non-inferiority of different treatments or doses might favor treatments which are ineffective or even harmful [41,42]. A phenomenon that has been termed ‘bio-creep’. The prevalence of bio-creep is a matter of ongoing research; [43] come to the conclusion, that it is not a major cause of concern in practice (at least on average). Awareness of the issue is nevertheless important and should even more underline the need to carefully think about which effect sizes are deemed meaningful, beyond simply comparing the results of studies with each other.

### 3.1 Possible misconceptions

Probability is not intuitive, and every statistical technique runs the risk of being misinterpreted. The techniques discussed in this article have great potential to improve statistical inferences, but it is important to prevent misinterpretations. When performing a null-hypothesis significance test, a non-significant result can not be used to conclude a meaningful effect is absent. To conclude this, one has to specify and test against whichever effect one defines to be ‘meaningful’. An equivalence test can be used to statistically *reject* effects as large or larger than the smallest effect size of interest, with a long-term error rate. It can not be used to conclude the effect is exactly 0, or to reject the presence of *any* effect. If we conclude statistical equivalence, we can reject the presence of effect sizes more extreme than the smallest effect size of interest with a known error rate, but we can not conclude the true effect is exactly zero – there might be a true but small effect. For this reason, conclusions based on equivalence tests must always specify the equivalence bounds that are used, and it is recommended to combine equivalence tests with null-hypothesis significance tests (which can also help to identify effects that are significant and equivalent, or practically insignificant differences). Thus, a statement such as ‘the difference was statistically equivalent to zero’ is imprecise, and a more precise interpretation is ‘we could reject effect sizes more extreme than the equivalence bounds of *−*0.4 and 0.4’.

When calculating the posterior distribution in Bayesian statistics, a prior is combined with the observed data. Any statements about the posterior distribution are not just based on the data, but also conditional on the model. The model includes the prior distributions which can be chosen rather freely. The prior distribution may represent a researchers beliefs prior to observing the data, but can also be used to regularise estimates or incorporate information from previous studies. It is thus important to explicitly state the model setup and provide a justification for the choice of a prior distributions when using Bayesian estimation. As with other measures of uncertainty such as confidence intervals, Bayesian credibility intervals are not guaranteed to contain true parameter values. The credible intervals contain values which are deemed credible based on the prior and the observed data with a specified posterior probability. Finally, when calculating Bayes factors, it is important to realize that they provide relative evidence for two specified models. A Bayes factor can indicate strong support for a null model relative to an alternative model, but both models can be wrong. The Bayes factor gives a relative indication of whether the data is more in line with the null-model or the alternative model.

### 3.2 Differences between inferential frameworks

All statistical methods give rise to probabilistic inferences. Rare events happen, and unlikely outcomes can be observed. Probabilistic methods can never be used to know with certainty that an effect is present or absent. Thus, none of the statistical techniques presented in this paper are capable of *proving* the null. After analyzing their data, researchers might be tempted to conclude ‘there was no effect’, but none of the statistical approaches discussed here allow for such a conclusion. It is important to understand the questions that the different statistical techniques described in this article provide an answer to.

Equivalence tests are used to make dichotomous conclusions to guide behavior, while controlling error rates in the long run. The goal of such a test is to reject the presence of effects large enough to matter, without being wrong too often. Any single study might lead to an incorrect conclusion, but theories that are correct should make predictions that are confirmed with expected error rates in lines of research. Although single studies are never sufficient to draw strong conclusions in science, this idea is especially central in frequentist statistics.

Bayesian statistics focus more strongly on quantifying beliefs or making statements about which values are deemed credible. In the case of Bayesian estimation, the focus lies on allocating credibility to parameter values (such as effect sizes or differences between groups), which can result in statements about degrees of belief. In the case of Bayes factors, the focus lies on quantifying the rational change in belief in a null-model or an alternative model, which is also termed *statistical evidence* [44]. Although there are many different flavors of Bayesian statistics, a strength of these approaches lies in drawing conclusions that incorporate pre-existing information in statistical inferences. Whether quantified beliefs or any other statistical inference corresponds with reality depends on how accurate model assumptions are. This is relevant for Bayesian models and the chosen prior distributions as well as for model assumptions in frequentist statistics.

In Bayesian estimation the prior can be used to shrink or regularise parameter estimates. Through Bayes’ theorem, priors provide an automatic way to implement shrinkage in a statistical model. Especially in small samples and more complex models, this avoids overfitting the data and can lead to better estimates for out-of-sample inferences and predictions [45]. With more data parameter estimates become more precise and the prior has less influence on the posterior distribution, thus providing less shrinkage as is desirable in most models. Finally, the Bayesian approach to statistical modelling is very versatile and can be used even in complex models such as hierarchical generalized models. Bayesian hierarchical or multilevel models are particularly useful in clinical research, for example, when using clustered samples or repeated measurements [45–47].

## 4. Conclusions

Null hypothesis significance testing has been critised because it is often misused and misunderstood [48]. Researchers who only rely on null-hypothesis significance tests limit themselves in only asking the question whether the null-hypothesis can be rejected. By adding statistical techniques such as equivalence testing, Bayesian estimation, and Bayes factors to ones repertoire, researchers can substantially improve the inference they can draw from null-effects by asking more relevant questions. Being able to demonstrate the absence of effects is important in all major approaches to philosophy of science [49]. When researchers only publish scientific findings that statistically reject null effects, the scientific literature is biased, which hinders the accumulation of scientific knowledge [5,6]. By using statistical approaches that can provide informative conclusions about null effects, researchers might not be able to ‘prove the null’, but they can substantially improve their statistical inferences about null-effects.

## Conflict of interest disclosure

No conflicts of interests are reported.

## Footnotes

^1^ All data for the example study, along with analysis scripts for R [1], JASP [2], and jamovi [3], and the scripts used to create this manuscript and the figures in it are available at https://osf.io/wptju/.

^2^The study design and analysis plan used herein is simplified for illustrative purposes. Practitioners might in reality consider a multilevel analysis to better account for different sources of variation [10]. The general recommendations in this paper also apply to more complex models.

^3^Ideally, the alpha level is set based on a cost-benefit analysis of Type 1 and Type 2 errors, see [11].

^4^The scripts for generating the simulated samples, including the chosen population parameters (which are usually unknown to the researcher), are included in the accompanying OSF repository.

^5^Note, however, that in some practical cases frequentist confidence intervals and Bayesian credibility intervals yield the same range of values [30].

^6^The subscript in *BF01* specifies the relative evidence for the null compared to the alternative, but a Bayes Factor can also be expressed as the relative evidence for the alternative compared to the null, or BF_10_ = 1/4.2 = 0.24
